# Supplementary material for: EnzML: multi-label prediction of enzyme classes using InterPro signatures
Source: BMC Bioinformatics. 2012 Apr 25;13:61. doi: 10.1186/1471-2105-13-61 (PMC3483700; doi:10.1186/1471-2105-13-61)
Supplement: Addtional file 5 — The Java code to format the data files, evaluate and predict. The file enzml_java_code.tar.gz contains the Java code used to format database data to ARFF and XML formats, to execute cross and train-test (jackknife) evaluations and to record evaluation results to database. More information is included in the readme.txt file and the Javadoc files. The code can be used with a MySQL database. To use a different database software, other JDBC drivers might be required. [file 1471-2105-13-61-S5.gz › java_code/utils/doc/uk/ac/ed/inf/utils/FileUtils.html]

FileUtils


---


|  |  |  |
| --- | --- | --- |
| **Overview** \| **Package** \| **Class** \| **Use** \| **Tree** \| **Deprecated** \| **Index** \| **Help** | |  |
| **PREV CLASS**   **NEXT CLASS** | **FRAMES**    **NO FRAMES**     **All Classes** |  |
| SUMMARY: NESTED \| FIELD \| CONSTR \| METHOD | DETAIL: FIELD \| CONSTR \| METHOD |  |


---


## uk.ac.ed.inf.utils Class FileUtils

```
java.lang.Object
  uk.ac.ed.inf.utils.FileUtils
```

---

``` public class FileUtils extends java.lang.Object ```

Utils to read, write files etc.

**Version:**
:   24-Mar-2007

**Author:**
:   ldeferra

---

**Constructor Summary**

`FileUtils()`


| **Method Summary** | |
| --- | --- |
| `static void` | `appendPropertiesToFile(java.io.File file, java.util.Properties properties)` |
| `static void` | `appendToFile(java.io.File file, java.lang.String text)`             Append text to file |
| `static void` | `appendToFile(java.lang.String filePath, java.lang.String text)`             Append text to file |
| `static void` | `appendToFileAndPrint(java.lang.String filePath, java.lang.String text)`             Append print text to screen (system.out) and append it to file. |
| `static boolean` | `canReadFile(java.io.File file)`             Tests wether the file can be read |
| `static boolean` | `canReadFile(java.lang.String path)`             Tests whether the file can be read |
| `static boolean` | `canReadParent(java.lang.String path)`             Checks whether a file directory is readable. |
| `static boolean` | `canWriteFile(java.io.File file)`             Tests whether the file can be written |
| `static boolean` | `canWriteFile(java.lang.String path)`             Tests wether the file can be written |
| `static boolean` | `canWriteParent(java.lang.String path)`             Checks whether a file directory is writable. |
| `static boolean` | `deleteFile(java.lang.String fileName)`             Delete a file |
| `static void` | `fileNotWritablePopUp(java.lang.String filePath)`             Error Pop-up: file not writable |
| `static java.lang.String[][]` | `fileToArrayOfArrays(java.lang.String fileName, java.lang.String regExpOfSeparator, boolean discardFirstLine)`             Reads a special-character-separated file (eg comma or tab separated) and creates an array of rows-columns. |
| `static java.util.ArrayList<java.lang.String>` | `fileToArrayOfLines(java.io.File file)`             Reads a file and adds the lines to an array, adapted from: http://www.computing.net/programming/wwwboard/forum/12276.html |
| `static java.util.ArrayList<java.lang.String>` | `fileToArrayOfLines(java.lang.String filePath)`             Reads a file and adds the lines to an array, adapted from: http://www.computing.net/programming/wwwboard/forum/12276.html |
| `static java.util.ArrayList<java.lang.String>` | `fileTokensToArray(java.lang.String fileName, java.lang.String regexp)`             Reads a file and adds the tokens (identified by regular expressions) to an array (e.g. |
| `static void` | `fromArrayOfArraysToFile(java.util.ArrayList<java.util.ArrayList<java.lang.String>> lists, java.lang.String fileName, java.lang.String regExpOfSeparator)`             Writes a special-character-separated file (eg comma or tab separated) and fills it from an array of rows-columns. |
| `static java.io.BufferedReader` | `fromFilePathToBufferedReader(java.lang.String filePath)`             From file path to bufferedReader |
| `static java.lang.String[]` | `getDirectoryFiles(java.lang.String directoryPath)`             Gets the list of files in a directory From http://www.faqs.org/docs/javap/c10/s2.html |
| `static java.lang.String` | `getDirectoryPath(java.lang.String path)`             Get the parent of a full file path (directory + filename) |
| `static java.lang.String` | `getFileNameFromDirectory(java.lang.String directoryPath, java.lang.String pattern)`             Get the first file name in the directory matching the given pattern string. |
| `static java.lang.String` | `getFileNameFromFullPath(java.lang.String path)`             Get the filename from a full file path (directory + filename) |
| `static long` | `getFileSizeInKb(java.lang.String filePath)`             Get the file size in Kb |
| `static java.util.zip.GZIPInputStream` | `getGunzipInputStream(java.io.BufferedInputStream source)` |
| `static java.io.File` | `getPotentialDirectory(java.io.File file)`             Get a directory out of a path |
| `static java.lang.String` | `getStringFromBufferedReader(java.io.BufferedReader reader)` |
| `static java.lang.String` | `getStringFromFile(java.lang.String filePath)`             Get all the text in a file as a single string |
| `static java.util.zip.GZIPInputStream` | `gunzipFile(java.io.File inputFile)` |
| `static int` | `gunzipFile2(java.io.File file_input, java.io.File dir_output)`             Gunzip the input archive. |
| `static void` | `main(java.lang.String[] args)`             Main method for testing |
| `static void` | `printAllPaths(java.io.File file)`             Test method to print all absolute/relative paths of a file |
| `static void` | `printFromReader(java.io.BufferedReader reader)`             Prints all lines from a reader. |
| `static void` | `writeToFile(java.lang.String filePath, java.lang.String text)` |

**Methods inherited from class java.lang.Object**

`equals, getClass, hashCode, notify, notifyAll, toString, wait, wait, wait`

**Constructor Detail**

### FileUtils

```
public FileUtils()
```


**Method Detail**

### appendPropertiesToFile

```
public static void appendPropertiesToFile(java.io.File file,
                                          java.util.Properties properties)
```

---


### appendToFile

```
public static void appendToFile(java.io.File file,
                                java.lang.String text)
```

:   Append text to file

    :   **Parameters:**: `file` - a File object: `text` - a text string **Throws:**: `java.io.IOException`

---


### appendToFile

```
public static void appendToFile(java.lang.String filePath,
                                java.lang.String text)
```

:   Append text to file

    :   **Parameters:**: `filePath` - the full path to an existing file: `text` - a text string

---


### appendToFileAndPrint

```
public static void appendToFileAndPrint(java.lang.String filePath,
                                        java.lang.String text)
```

:   Append print text to screen (system.out) and append it to file.

    :   **Parameters:**: `filePath` - the full path to an existing file: `text` - a text string

---


### canReadFile

```
public static boolean canReadFile(java.io.File file)
```

:   Tests wether the file can be read

---


### canReadFile

```
public static boolean canReadFile(java.lang.String path)
```

:   Tests whether the file can be read

---


### canReadParent

```
public static boolean canReadParent(java.lang.String path)
```

:   Checks whether a file directory is readable. For example for file
    /home/user/file.txt, it checks if /home/user/ is readable

    :   **Parameters:**: `path` - directory path + filename **Returns:**: true if the path before the last slash is readable

---


### canWriteFile

```
public static boolean canWriteFile(java.io.File file)
```

:   Tests whether the file can be written

---


### canWriteFile

```
public static boolean canWriteFile(java.lang.String path)
```

:   Tests wether the file can be written

---


### canWriteParent

```
public static boolean canWriteParent(java.lang.String path)
```

:   Checks whether a file directory is writable. For example for file
    /home/user/file.txt, it checks if /home/user/ is writable

    :   **Parameters:**: `path` - directory path + filename **Returns:**: true if the path before the last slash is writable

---


### deleteFile

```
public static boolean deleteFile(java.lang.String fileName)
```

:   Delete a file

    :   **Parameters:**: `filePath` - the full path to a file **Returns:**: true if the file was deleted

---


### fileNotWritablePopUp

```
public static void fileNotWritablePopUp(java.lang.String filePath)
```

:   Error Pop-up: file not writable

---


### fileToArrayOfArrays

```
public static java.lang.String[][] fileToArrayOfArrays(java.lang.String fileName,
                                                       java.lang.String regExpOfSeparator,
                                                       boolean discardFirstLine)
```

:   Reads a special-character-separated file (eg comma or tab separated) and
    creates an array of rows-columns. Each column contains the column data
    elements

    :   **Parameters:**: `fileName` - the full path to the file to be read: `regExpOfSeparator` - the regular expression separating the tokens in the line (e.g. a comma for comma separated values files) **Returns:**: an array of arrays in which each element is a token from the file (row by columns)

---


### fileToArrayOfLines

```
public static java.util.ArrayList<java.lang.String> fileToArrayOfLines(java.io.File file)
```

:   Reads a file and adds the lines to an array, adapted from:
    http://www.computing.net/programming/wwwboard/forum/12276.html

    :   **Parameters:**: `file` - the file to read from **Returns:**: the array containing the file lines

---


### fileToArrayOfLines

```
public static java.util.ArrayList<java.lang.String> fileToArrayOfLines(java.lang.String filePath)
```

:   Reads a file and adds the lines to an array, adapted from:
    http://www.computing.net/programming/wwwboard/forum/12276.html

    :   **Parameters:**: `filePath` - the path of the file to read from **Returns:**: the array containing the file lines

---


### fileTokensToArray

```
public static java.util.ArrayList<java.lang.String> fileTokensToArray(java.lang.String fileName,
                                                                      java.lang.String regexp)
```

:   Reads a file and adds the tokens (identified by regular expressions) to
    an array (e.g. for flat files with a long list of values \_without\_ any
    new-line) adapted from:
    http://www.computing.net/programming/wwwboard/forum/12276.html

    :   **Parameters:**: `fileName` - the path of the file to read from **Returns:**: array with the string tokens

---


### fromArrayOfArraysToFile

```
public static void fromArrayOfArraysToFile(java.util.ArrayList<java.util.ArrayList<java.lang.String>> lists,
                                           java.lang.String fileName,
                                           java.lang.String regExpOfSeparator)
                                    throws java.io.IOException
```

:   Writes a special-character-separated file (eg comma or tab separated) and
    fills it from an array of rows-columns. Each column contains the column
    data elements

    :   **Parameters:**: `fileName` - the file to be read: `regExpOfSeparator` - the regular expression separating the tokens in the line (e.g. a comma for comma separated values files) **Throws:**: `java.io.IOException`

---


### fromFilePathToBufferedReader

```
public static java.io.BufferedReader fromFilePathToBufferedReader(java.lang.String filePath)
```

:   From file path to bufferedReader

    :   **Parameters:**: `filePath` - **Returns:**: a BufferedReader **Throws:**: `java.io.FileNotFoundException`

---


### getDirectoryFiles

```
public static java.lang.String[] getDirectoryFiles(java.lang.String directoryPath)
```

:   Gets the list of files in a directory From
    http://www.faqs.org/docs/javap/c10/s2.html

    :   **Parameters:**: `directoryPath` - **Returns:**: a list of files in the directory

---


### getDirectoryPath

```
public static java.lang.String getDirectoryPath(java.lang.String path)
```

:   Get the parent of a full file path (directory + filename)

    :   **Parameters:**: `path` - a full file path (directory + filename) **Returns:**: the parent directory full path

---


### getFileNameFromDirectory

```
public static java.lang.String getFileNameFromDirectory(java.lang.String directoryPath,
                                                        java.lang.String pattern)
```

:   Get the first file name in the directory matching the given pattern
    string.

    :   **Parameters:**: `pattern` - a regular expression **Returns:**: the path of the first file matching the pattern

---


### getFileNameFromFullPath

```
public static java.lang.String getFileNameFromFullPath(java.lang.String path)
```

:   Get the filename from a full file path (directory + filename)

    :   **Parameters:**: `path` - a full file path (directory + filename) **Returns:**: the filename (string after the last slash or backslash)

---


### getFileSizeInKb

```
public static long getFileSizeInKb(java.lang.String filePath)
```

:   Get the file size in Kb

---


### getGunzipInputStream

```
public static java.util.zip.GZIPInputStream getGunzipInputStream(java.io.BufferedInputStream source)
```

---


### getPotentialDirectory

```
public static java.io.File getPotentialDirectory(java.io.File file)
```

:   Get a directory out of a path

---


### getStringFromBufferedReader

```
public static java.lang.String getStringFromBufferedReader(java.io.BufferedReader reader)
```

---


### getStringFromFile

```
public static java.lang.String getStringFromFile(java.lang.String filePath)
```

:   Get all the text in a file as a single string

    :   **Parameters:**: `filePath` - **Returns:**: all the text in the file as a single string **Throws:**: `java.io.IOException`

---


### gunzipFile

```
public static java.util.zip.GZIPInputStream gunzipFile(java.io.File inputFile)
```

---


### gunzipFile2

```
public static int gunzipFile2(java.io.File file_input,
                              java.io.File dir_output)
```

:   Gunzip the input archive. Send the output to the directory specified by
    dir\_output. Assumes that the input file name ends with ".gz" from
    http://www
    .particle.kth.se/~lindsey/JavaCourse/Book/Part1/Supplements/Chapter09
    /compressDecompress.html

---


### main

```
public static void main(java.lang.String[] args)
```

:   Main method for testing

    :   **Parameters:**: `args` - **Throws:**: `java.sql.SQLException`

---


### printAllPaths

```
public static void printAllPaths(java.io.File file)
                          throws java.io.IOException
```

:   Test method to print all absolute/relative paths of a file

    :   **Parameters:**: `file` - **Throws:**: `java.io.IOException`

---


### printFromReader

```
public static void printFromReader(java.io.BufferedReader reader)
                            throws java.io.IOException
```

:   Prints all lines from a reader.

    :   **Parameters:**: `reader` - a buffered reader object (taking text from a file, or URL etc.) **Throws:**: `java.io.IOException`

---


### writeToFile

```
public static void writeToFile(java.lang.String filePath,
                               java.lang.String text)
```


---


|  |  |  |
| --- | --- | --- |
| **Overview** \| **Package** \| **Class** \| **Use** \| **Tree** \| **Deprecated** \| **Index** \| **Help** | |  |
| **PREV CLASS**   **NEXT CLASS** | **FRAMES**    **NO FRAMES**     **All Classes** |  |
| SUMMARY: NESTED \| FIELD \| CONSTR \| METHOD | DETAIL: FIELD \| CONSTR \| METHOD |  |


---
